# Pancreatic ductal adenocarcinoma can be detected by analysis of volatile organic compounds (VOCs) in alveolar air

**DOI:** 10.1186/s12885-018-4452-0

**Published:** 2018-05-04

**Authors:** Andrea Princivalle, Lorenzo Monasta, Giovanni Butturini, Claudio Bassi, Luigi Perbellini

**Affiliations:** 1 Department of Diagnostics and Public Health – Occupational Medicine Unit, University of Verona, Policlinico Borgo Roma, Piazzale LA Scuro 10, 37134 Verona, Italy; 2 Epidemiology and Biostatistics Unit, Institute for Maternal and Child Health IRCCS “Burlo Garofolo”, Trieste, Italy; 3 Department of Surgery, Pederzoli Hospital, Peschiera del Garda, Verona, Italy; 4 Department of Surgery, Pancreas Inst, Gen Surg Unit B -Verona University Hospital, Verona, Italy

**Keywords:** Alveolar air, Volatile organic compounds, Pancreatic adenocarcinoma, IMR-MS, LASSO logistic regression

## Abstract

**Background:**

In the last decade many studies showed that the exhaled breath of subjects suffering from several pathological conditions has a peculiar volatile organic compound (VOC) profile. The objective of the present work was to analyse the VOCs in alveolar air to build a diagnostic tool able to identify the presence of pancreatic ductal adenocarcinoma in patients with histologically confirmed disease.

**Methods:**

The concentration of 92 compounds was measured in the end-tidal breath of 65 cases and 102 controls. VOCs were measured with an ion-molecule reaction mass spectrometry. To distinguish between subjects with pancreatic adenocarcinomas and controls, an iterated Least Absolute Shrinkage and Selection Operator multivariate Logistic Regression model was elaborated.

**Results:**

The final predictive model, based on 10 VOCs, significantly and independently associated with the outcome had a sensitivity and specificity of 100 and 84% respectively, and an area under the ROC curve of 0.99. For further validation, the model was run on 50 other subjects: 24 cases and 26 controls; 23 patients with histological diagnosis of pancreatic adenocarcinomas and 25 controls were correctly identified by the model.

**Conclusions:**

Pancreatic cancer is able to alter the concentration of some molecules in the blood and hence of VOCs in the alveolar air in equilibrium. The detection and statistical rendering of alveolar VOC composition can be useful for the clinical diagnostic approach of pancreatic neoplasms with excellent sensitivity and specificity.

**Electronic supplementary material:**

The online version of this article (10.1186/s12885-018-4452-0) contains supplementary material, which is available to authorized users.

## Background

The use of exhaled air in clinical practice had acquired significant prominence by the end of the 80s, when this biological material started to be used for the diagnostics of poisoning from volatile substances [1] or for the biological monitoring of professional exposures to solvents [2, 3].

In 1985 for the first time the possibility of identifying the presence of lung cancer through the examination of exhaled air [4] was pointed out. In subsequent phases Phillips et al. [5] made an analogous research using less complex equipment and confirmed the possibility of recognizing the presence of lung neoplasms using alveolar air. The same group of Authors stated that they had been able to observe 3481 different volatile organic compounds (VOCs) in exhaled air. Among them, 1753 had a positive alveolar gradient which suggests a prevalent endogenous or metabolic origin. The other 1728 had a negative alveolar gradient suggesting a prevailingly exogenous origin [6].

The same team published some results concerning patients with breast cancer [7]. Additionally, they broadened and deepened the analysis on lung neoplasms [8].

Theoretically, every important biological variation of the organism should come with significant biochemical changes and with interferences to the functionality of the immune defence system.

In fact, some modifications in the profile of the products present in the exhaled air were recorded in various clinical conditions: hepatic steatosis [9] active tuberculosis [10] schizophrenia [11], heart transplant [12] and cystic fibrosis [13, 14].

Some changes in the profile of the products exhaled have been observed in pregnant women affected by preeclampsia compared to those with a normal pregnancy [15].

The possibility of identifying subjects affected by different kinds of neoplasms requires the use of various analytic methods with very different characteristics. Mazzone et al. [16] prepared a “colorimetric sensor array” which by examining the exhaled air of patients affected by lung neoplasm, permits us to distinguish patients from controls with considerable sensitivity and specificity. Some types of “artificial odour sensors” allowed Taivans et al. [17] to distinguish patients with various lung pathologies (bronchial asthma and chronic obstructive pulmonary disease) including lung neoplasms, from control subjects. Peng et al. [18] used gold nanoparticle sensors to analyse the VOCs in the exhaled air of patients affected by lung cancers, or rectal colon, or prostate disorders, to separate them from control subjects. Using a “proton transfer reaction mass spectrometry”, Bajtarevic et al. [19] were able to detect some revealing differences in the quality of the exhaled air of subjects affected by lung cancer if compared to control subjects.

In this multifaceted frame of researches, we analysed the alveolar air in a group of patients affected by pancreatic ductal adenocarcinoma (PDA) cytohistologically confirmed and in a group of control subjects using an ion molecule reaction mass spectrometry (IMR-MS). The method we applied for the analysis of the alveolar air samples has been used just in few studies, but with good results [9, 20]; nevertheless it is frequently used for the analysis of environmental pollutants. In our opinion this method provides very interesting data in particular if they are associated to an appropriated statistical elaboration. The goal of the present study was to examine whether each profile of the analysed VOCs could permit us to identify an algorithm discriminating the two groups of subjects, able to become a novel safe, not invasive and easy method to diagnose PDA.

## Methods

### Subjects

In this study the cases enrolled were subjects admitted to the Pancreatic Unit of the Surgical Department at the Verona University Hospital between 2008 and 2013. The inclusion criteria were the cytohistological diagnosis of PDA, and age higher than 18 years. For each patient we collected: personal and medical history, smoking patterns, alcohol consumption, oncologic marker CA19-9, and the report of the final cytological or histological exam. The diagnosis of pancreatic ductal adenocarcinoma (PDA) is not easy and certainly difficult at an early stage. Sometime the PDA is found during routine health checks and is not accompanied by any clinical symptoms. Sometime some clinical symptoms such jaundice or intestinal disorders require thorough diagnostic tests which lead to the diagnosis of PDA. Often only histological results allow a sure diagnosis. We involved patients with diagnosis of PDA and planned to undergo surgery without any recent chemotherapy in order to verify possible changes in the composition of the alveolar air; the neoplasm was therefore not in a too advanced stage, but in some cases not in the early stage neither. Patients involved in this study were planned for the surgical intervention and alveolar air samples were collected 1-3 days before surgery in order to avoid any interference with VOCs in alveolar air related to drugs used in the operating room or to the stress related of surgical intervention. Controls were recruited during hospital medical inspections. It is difficult to identify “perfectly healthy control subjects” and with the same age as patients with pancreatic neoplasm; we selected people hospitalized and submitted to some clinical tests that did not detect any neoplasm or other important diseases. Many control subjects were hospitalized patients planned for slight surgical interventions such venous or hemorrhoidal varices, inguinal hernias etc. Other control subjects were outpatients selected among people periodically checked for slight respiratory disorders (e.g., non-allergic rhinitis, mild bronchial asthma) hypertension or psychological disorders but in good general health conditions. Moreover, even the same pathologies were also recorded in several patients with pancreatic cancer. Subjects with previously diagnosed neoplasms, or with significant pathologies, such as multiple sclerosis or other pathologies of the central nervous system, were excluded from the control group. Patients provide their alveolar samples in the morning, fasted, to avoid the possible interferences of food or its metabolites on the VOCs profile. Also controls gave their alveolar air samples always in the morning, fasted or more than 2 h away from meals (for outpatients). All subjects involved provided written consent to their participation in the research.

In a second phase, to validate the predictive model, we analysed breath samples on a further group of fifty subjects, 24 of them with PDA and 26 as controls: the same inclusion criteria above described (age: between 40 and 75 years) were adopted: alveolar air samples were collected 1-3 days before surgery in patients with PDA histologically confirmed. Control subjects were selected among outpatients periodically checked for moderate respiratory disorders or for work related psychological disorders, not affected by any neoplasm and in good general health conditions. Their alveolar air samples were collected in the morning 2 h away from meals.

### Sampling of alveolar air

Subjects were asked to make one deep exhalation inside a hand-device called Bio-VOC breath sampler® (Markes International Ltd., Rhondda Cynon Taff, UK), which is in essence a special 250 ml air syringe; it has got a valve in order to avoid any re-breathing phase by the subject, especially at the end of the blow, and the exhaled air follows a one way path. It was originally designed for air sampling discharged into sorbent tubes or for direct discharge on read out instruments.

While the subject was exhalating into the Bio-Voc breath sampler, a test tube, or vial was put at the other side of the sampler, to collect the exhaled air. The vial is described as a 20 ml glass tube with wide bordered opening, formerly sterilized and kept at 80 °C for at least 24 h, to avoid presence of environmental pollutants. The whole exhalation was channelled into the test tube, in order to further “clean” from possible environmental VOC. At the end of the exhalation, the vial (now containing the last part of the exhaled air, the alveolar air) was closed with a Teflon septum (PTFE) and hermetically sealed with an aluminium ring. A sample of environmental air in the same room was also taken into a 20 ml vial: air in the vial was drawn with a 100 ml manual pump to allow environmental air to fill it. In this case too, the vial was closed and sealed.

The test tubes were kept at − 20 °C until the analysis. The quality of the collection of the alveolar air was evaluated by the concentration of CO_2_ registered in the analysis: samples with less than 2% CO_2_ were considered as incorrectly collected, and were excluded from the statistical analysis.

### Equipment

The analysis of the VOCs was performed by using the AirSense analyser and the V&F-auto sampler (V&F medical development GmbH, Absam, Austria) as described by Hornuss et al. [20] and Netzer et al. [9]. This equipment uses a combination of two integrated Mass spectrometer (MS) systems consisting of a conventional MS capable of fast CO_2_ tracing controlling a second, highly sensitive MS for measuring the VOCs [21]. The AirSense consists of a highly sensitive MS with a soft ionization unit. The ionization of sample gas is performed through soft ionization by charge exchange between a primary ion (e. g. Xe^+^; 12,1 eV) and the molecule to be analysed. Primary ions are produced by electron impact in a closed ion source. This technology allows interference-free detection of molecules, which would not be possible by means of standard ionization techniques with energetically fast electrons (at least 70 eV) due to fragmentation and ionization (mass identity). The primary ions are focused by high frequency fields in an octopole separator and guided to the charge exchange cell. Through mass separation, contamination of the spectrum from source gases, as well as O_2_ and N_2_, is avoided and thus the sensibility of the system rises considerably. In the charge exchange, cell ions meet the sample gas which is ionized through charge exchange reactions (Ion Molecule Reaction). Here too, ions are focused by high frequency fields and led to the actual high resolution mass filter.

Each alveolar air sample is transferred to the mass spectrometer through the V&F-auto sampler which maintains the biological sample at 65 °C for 60 min before the transfer. In few seconds the concentration of several VOCs in the alveolar air sample is obtained.

The calibration of the mass spectrometer was performed by using various calibration mixtures containing CO_2_ and O_2_ at 10 and 5% respectively (from Messer Italia spa, Settimo Torinese, Italy). The measured gas compounds were given as absolute concentrations (ppb) and volume percent for CO_2_ and O_2_. Twelve other products were directly calibrated: methanol, acetonitrile (ACN), ethanol, methyl ethyl ketone (MEK), acetone, isoprene, n-propanol, benzene, toluene, n-heptane, ammonia (NH_3_) and sulphur dioxide (SO_2_). Other VOCs were indirectly calibrated to the sensitivity of one directly calibrated component (benzene) and named with “M” followed by the molecular weight of compounds detected. This nomenclature is maintained in the following paper’s text and in figures and tables too.

This concept represents a semi quantitative calibration procedure that is widely used in (multicomponent) analytical devices.

### Statistics

Considering the large number of independent variables involved in the analysis, we decided to base the elaboration of the predictive model on a LASSO (Least Absolute Shrinkage and Selection Operator) logistic regression (LLR) [22, 23]. The LASSO is a penalized estimation method which avoids overfitting, caused by to collinearity or high-dimensionality of independent variables, through the shrinking of the estimated regression coefficients. A tuning parameter λ controls the amount of shrinkage applied to the estimates. The shrinkage of some coefficients to zero reduces the number of covariates in the final model.

A 50-fold cross-validation was applied to all steps of the LLR, and independent variables (molecules) were standardized to allow optimal penalization.

In detail, we adopted a slightly modified version of the iterated LLR as described by Huang and colleagues [24]. First, we used an LLR to reduce the number of variables in the model, eliminating all variables if coefficients were 0 and ignoring their coefficients if these were > 0. Second, we included the remaining variables in a two-steps iterated LLR [24]: a first LLR to generate penalized weights to be used in an adaptive LLR [25]. Penalized weights were calculated as inverse logistic regression coefficients. For this last regression, confidence intervals of the regression coefficients were calculated with a 10-fold iterated Bootstrap procedure.

Descriptive analyses were carried out with Stata/IC 11.2 (StataCorp LP, College Station, TX, USA). Logistic regressions were carried out with R version 2.15.1 (The R Foundation for Statistical Computing, Vienna, Austria) and R packages “penalized” (Goeman JJ. Penalized R package, version 0.9-42) and “polywog” (Kenkel B, Signorino CS. Bootstrapped Basis Regression with Oracle Model Selection, version 0.2-0).

## Results

In this study we enrolled 219 subjects (mean age 52 years, range 21-83) who signed an informed consent form. Among them, 144 were controls (mean age 47, range 21-78) and 75 were patients suffering from PDA (mean age 62, range 23-83).

All subjects were able to carry out the air sampling without difficulties, after a quick and easy explanation. Out of the 75 patients with PDA, we recorded positivity for the marker Ca19.9 in 49 patients.

In order to homogenize ages between cases and controls, however, we restricted the sample to cases and controls with ages ≥40 and ≤ 75. We, thus, excluded one 23 year old patient with ductal adenocarcinoma, 41 controls of < 40 years of age, one 78 year old control and 9 cases of ductal adenocarcinoma with > 75 years of age. A description of the restricted sample of 167 subjects (102 controls, 65 cases with ductal adenocarcinoma,) is presented in Table 1. We found no significant difference in terms of sex distribution between controls and the outcome group. Despite the age restriction, however, a significant difference was found between age of controls and age of patients suffering from PDA.

**Table 1:** Alveolar air and pancreatic neoplasms: description of the sample of cases and controls of ages ≥40 and ≤ 75, n.167*

	Controls (n.102)	Ductal Adenocarcinomas (n.65)
Gender		
- males	51 (50%)	36 (55%)
- females	51 (50%)	29 (45%)
Age		
- mean (SD)	53 (9)	61 (8)
- median (IQR)	51 (45-61)	61 (55-68)**
Preexisting or previous diseases		
Arterial hypertension	12	10
diabetes mellitus	6	13
Other previous and resolved neoplasms	3	11
other pancreatic diseases	2	//
other pathologies	11	6

*SD* standard deviation, *IQR* inter-quartile range

*Differences between controls and outcome categories were calculated with Fisher exact test for dichotomous independent variables and with non-parametric Mann-Whitney rank sum test for continuous variables not distributed normally. Only significant differences (*p* < 0.05) are reported in the table

**Mann-Whitney: *p* = 0.000

In Table 2 we report some descriptive parameters that summarize the results of the measurement for the VOCs, for which we prepared a scaling curve, in samples of both environmental air and alveolar air of control subjects. The concentration ranges (in ppb) found in the international literature confirm that our results are reliable and comparable with those registered by other authors**,** both for the environmental concentration and for alveolar one [21, 26–31]. Moreover, indoor air concentration of SO_2_ and NH_3_ are similar to those found by Leaderer et al. [32] and Salem et al. [33].

**Table 2:** Environmental and alveolar concentration of products for which we prepared a specific calibration. Data are expressed in ppb and regard 219 samplings both for environmental and alveolar concentrations

	Enviromental Concentration				Alveolar Concentration			
	Mean	Median	range	5-95th percentile	Mean	Median	range	5-95th percentile
Methanol	654	633	267-1789	323-1166	537	492	259-3021	294-841
Acetonitrile	196	129	34-1820	48-802	269	176	43-1840	66-1164
Ethanol	924	525	71-6244	121-3187	532	349	47-8668	88-1361
MEK	55	45	12-352	14-147	102	78	20-622	36-281
Acetone	217	181	52-2432	67-392	721	534	116-10,179	244-1676
Isoprene	20.8	18	5-262	8.9-34.4	160	149	17-428	63-292
n-Propanol	79	47	16-2093	20-172	98	80	31-619	45-194
Benzene	6.1	5.6	0.1-21	0.7-13	11.7	9.4	0.9-85.7	2.2-35.9
Toluene	5.2	4.8	0.1-22.1	1.1-11.7	5.8	4.8	0.6-25.9	1.6-14.5
n-Heptane	19.5	14.3	4.6-679.5	6.7-34.1	18.7	15.6	4.1-500.1	6-31.5
SO_2_	10.3	10.4	4.3-25.7	5.9-14.6	5.9	9.8	3.1-16.7	4.5-13.8
NH_3_	77	70	17-302	24-116	118	101	14-737	40-246

**Fig. 1:**
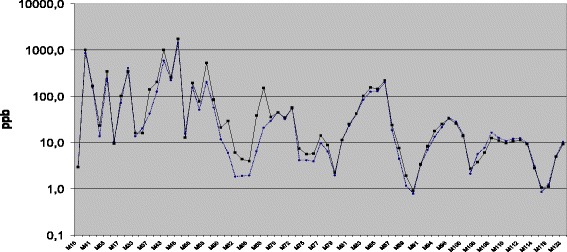
Alveolar (unbroken line) and environmental (dotted line) concentrations used for the statistics (70 groups of molecules): molecules which were significantly more present in the environmental air than in the alveolar air have been excluded (data are reported in ppb). The 22 molecules excluded were the following: M19, M26, M27, M28, M31, M32, M45, M48, M49, M73, M92, M97, M98, M102, M103, M104, M105, M116, M117, M18, M120 and M121

Figure 1 shows the profile of the products detected in the alveolar air of studied subjects (unbroken line) and in the environmental air (dotted line) in the consulting room where the patients and the control subjects were received. The concentration of the each molecule (in ppb) with specific molecular weight represents the median of 219 samples. The x-axis of the figure shows the series of the molecular weights of the analysed products. Data are shown after the deletion of the group of molecules with a significantly greater environment concentration, compared to the alveolar concentration: these molecules, partially absorbed by the human organism, are found in the alveolar air just in proportion to the amount kept at a pulmonary level. A higher alveolar concentration compared to the environmental one, suggests a biological-metabolic origin. Besides, the appearance of these molecules in the alveolar air is the expression of their partial positive pressure in the blood, compared to the environmental air, suggesting a tendency of the organism to eliminate them through the exhaled air. They are 70 molecules, among which are: acetone, acetonitrile, n-heptane, isoprene, MEK, NH_3_, SO_2_, toluene and benzene, for which we had prepared a specific calibration.

**Fig. 2:**
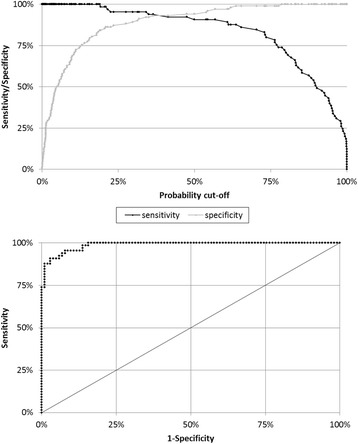
Sensitivity and specificity curves for the Ductal Adenocarcinoma Model and Receiver Operating Characteristic curve (area under ROC = 0.9879)

The first LLR was run with age, sex and all 70 VOCs. From the 50-fold cross-validation procedure we obtained a λ value of 1.867425. Applying this λ we obtained a model with 21 VOCs plus age: all other variables (49 VOCs and sex) had regression coefficients equal to zero and were thus discarded. The second LLR (LASSO followed by an Adaptive LASSO), which included age and the remaining 21 VOCs, produced a model based on age plus 10 VOCs (Table 3). This final model has an area under the ROC curve of 0.9879 (Fig. 2). This model is able to identify all cases (sensitivity = 100%) with only 16 false positives out of 102 (specificity = 84.3%). Otherwise, with a sensitivity of 95.4% (3/65 false negatives), specificity reaches 92.2% (8/102 false positives).

**Table 3:** Ductal Adenocarcinoma Model obtained from the first and second LASSO logistic regression (*n* = 167)

Variables	Regression Coefficients of the first LLR^a^	Regression Coefficients of the second LLR^b^	95% CI^b^
Intercept	−5.3255467964	−4.832765	−19.837 – + 4.153
Age	+ 0.1493804071	+ 0.119876	+ 0.032 – + 0.403
Ammonia	+ 0.0036286701	+ 0.001723	+ 0.000 – + 0.023
M34	−0.1699431526	− 0.102787	− 0.402 – + 0.000
M37	+ 0.0006675672	//	//
M42	+ 0.0009076002	//	//
M43	+ 0.0007153015	+ 0.002183	−0.006 – + 0.005
M44	+ 0.0047526254	+ 0.001295	+ 0.001 – + 0.051
M46	−0.0007407600	//	//
M60	−0.0003390278	//	//
Propanol	+ 0.0054942415	//	//
M62	−0.2138505207	−0.335321	−1.007 – − 0.077
Sulfur dioxide	− 0.2595054348	−0.384319	− 1.116 – − 0.132
M66	− 0.1459224298	//	//
M71	+ 0.0062530914	+ 0.012486	+ 0.000 – + 0.040
M74	−0.0120892660	−0.021012	− 0.159 – + 0.000
M75	− 0.0008072235	//	//
M88	−0.0142597155	//	//
M89	+ 0.2297944263	+ 0.426043	+ 0.536 – + 2.368
M91	−0.0286331109	//	//
M106	−0.0135291587	//	//
M111	+ 0.0926572905	//	//
M112	+ 0.0054448431	+ 0.023171	+ 0.000 – + 0.230

*CI* confidence interval, obtained from a 10-fold iteration Bootstrap procedure

^a^Regression Coefficients of the first LASSO logistic regression (2^nd^ column)

^b^Regression coefficients of the Ductal Adenocarcinoma Model obtained from the second, iterated LASSO logistic regression (3^rd^ and 4^th^ columns)

Table 4 reports the main statistical parameters of alveolar concentrations (expressed in ppb) of ten VOCs selected by the final model both for cancer patients and controls. Some VOCs have concentrations round a few ppb, but some others overtake thousands of ppb. In patients affected by PDA the median concentrations of Ammonia, M43, M71, M74, M89 and M112 are higher than in controls, while those of M34, M44, M62, Sulfur dioxide are lower.

**Table 4:** Main statistical parameters related to VOCs selected by the final predictive model to discriminate between 65 subjects with pancreatic ductal adenocarcinoma (PDA) and 102 controls (data in ppb)

	Ammonia	M34	M43	M44	M62	Sulfur dioxide	M71	M74	M89	M112
Control subjects										
Median	91.0	19.4	1025.7	7220.3	7.3	11.0	31.9	7.9	1.3	8.9
Geometrical Mean	88.8	18.7	1023.9	6576.8	7.5	10.4	36.1	9.4	1.5	9.6
Minimum value	13.8	9.4	288.8	2879.3	2.3	4.9	12.5	2.2	0.1	2.8
Maximum value	459.6	29.5	2697.8	14,011.8	41.7	14.5	186.9	383.5	15.2	152.6
Arithmetic mean	106.5	19.2	1087.5	6918.8	8.7	10.6	44.5	17.4	2.2	12.0
Standard deviation	72.9	4.0	397.8	2115.9	5.7	2.0	36.1	41.7	2.5	15.4
Patients with PDA										
Median	106.7	12.4	1195.7	6106.7	5.7	6.0	42.2	8.4	3.5	14.0
Geometrical Mean	118.2	12.8	1337.8	6141.6	6.3	6.9	41.2	9.6	3.4	14.5
Minimum value	33.4	8.5	402.4	3521.3	1.9	3.1	15.4	1.9	0.3	6.1
Maximum value	737.0	30.4	13,697.7	10,436.8	33.1	16.7	179.1	130.9	42.1	71.4
Arithmetic mean	136.7	13.3	1874.4	6371.2	7.7	7.7	49.4	16.2	5.7	16.7
Standard deviation	102.8	4.4	2109.5	1749.0	6.0	3.6	34.5	22.5	6.9	10.9

For an additional validation of the model we collected other alveolar air samples from new 50 subjects 40-75 years-old: 24 patients were admitted into our surgical department with histologically identified ductal adenocarcinoma and 26 were controls. Methods for VOC collection and their measurements were the same used for the other subjects. Table 5 reports the main statistical parameters of alveolar concentrations (expressed in ppb) of the ten VOCs selected by the model both for further new patients with PDA and controls. By using the same cut-off previously calculated, the model correctly classified 23 cases of 24, meaning a sensitivity of 95.8% and 25 controls of 26 with a specificity of 96.2%.

**Table 5:** Main statistical parameters related to VOCs related to the 50 subjects (26 controls and 24 cases) used for validation of the model itself

	Ammonia	M34	M43	M44	M62	Sulfur dioxide	M71	M74	M89	M112
Control subjects										
Median	101.5	17.7	849.5	5430.4	5.1	10.8	17.5	8.0	1.3	6.9
Geometrical Mean	101.0	18.6	873.9	5400.7	4.7	11.0	19.4	9.4	1.4	8.0
Minimum value	27.3	14.9	622.0	3755.8	2.1	9.0	12.4	4.5	0.1	5.1
Maximum value	359.1	24.2	1430.9	7560.7	11.0	14.8	46.0	106.9	14.1	73.5
Arithmetic mean	123.6	18.8	888.5	5513.7	5.1	11.0	20.5	13.7	1.9	10.1
Standard deviation	85.4	2.4	176.2	1134.5	2.2	1.1	7.9	20.1	2.6	13.1
Patients with PDA										
Median	185.7	9.8	1319.2	3154.1	11.3	6.3	21.6	31.0	5.1	19.5
Geometrical Mean	205.6	6.1	1518.1	2477.8	10.5	7.0	22.8	29.6	4.6	21.8
Minimum value	52.7	0.9	581.3	461.9	3.0	5.1	15.8	6.9	1.2	8.8
Maximum value	1193.3	18.5	5377.7	4964.1	29.7	38.7	38.0	302.7	11.4	58.2
Arithmetic mean	293.7	8.4	1811.5	2881.4	12.2	7.9	23.7	45.0	5.3	25.9
Standard deviation	293.7	5.0	1213.8	1337.4	6.8	6.6	6.7	60.9	2.9	15.7

## Discussion

The most relevant results of our study are:

i) In presence of PDA the normal VOCs profile in the exhaled air changes
ii) The profile of the VOCs has the potential to become a test to discriminate between subjects with ductal adenocarcinoma and subjects without the disease.

The adenocarcinoma of the pancreas is the most common pancreatic tumour (around 95%) and the fourth most common cause of death due to cancer in the USA It often has an unfavourable prognosis: considering all stages, the survival is respectively 25% at one year and 6% at 5 years [34]. Nevertheless the only possibility of treatment is the removal of small tumours formerly discovered [35].

Research for an early diagnosis is therefore a priority of extraordinary relevance. Unfortunately, at present the results have not been satisfactory [36], even though several approaches have been tried [37]. Serum CA19-9 represents the “gold standard” marker to verify the presence of a pancreatic cancer. The diagnostic sensitivity of the CA19-9 is 79% (70-90%), its specificity is 82% (68-91%), with a positive predictive value of 72% (41-95%) and the negative predictive value of 81% (65-98%) [38]. In addition, 10-15% of the people do not produce the CA19-9 because of the Lewis-negative genotype [39]*.* Besides, the serum levels of this indicator are high even in other benign and malignant pathologies [40].

Concerning 10 molecules we detected as important for the ductal Adenocarcinoma model only 5 are known: two of these are quantified and calibrated by IMR-MS in our equipment conditions; they are ammonia (M17) and sulphur dioxide (M64). Three compounds with MW 34 Da (Da), 43 and 44 Da, considering the electronic charge-discharge conditions of our instrument are hydrogen sulphide, acetyl group and acetaldehyde respectively. For the last five masses of ten, we did not find in the literature any report associating them to products already found by other Authors in the alveolar air samples of patients with any kind of cancer. Their chemical identification is not possible with the method we used and must be provided by other techniques (i.e. GC-MS, which however needs preconcentration of the sample). In order to better characterize these five unknown masses, we considered all the molecules with weights 62, 71, 74, 89 and 112 Da, reported by PubChem (http://pubchem.ncbi.nlm.nih.gov), or by the ChemSpider free-on-line database from the Royal Society of Chemistry (http://RSC.org; http://www.chemspider.com).

Among the matched molecules we excluded:

- Molecules of clear industrial or environmental origin (for example products containing chlorine atoms or fluorine, bromine, etc.);
- Molecules with ester linkage (as they are easily ionisable in the blood and they cannot be expelled with the alveolar air);
- Highly reactive molecules, which show instability in the biological matrix (i.e. free radicals);
- Molecules with a boiling point above 150 °C, with a high steam pressure (above 20 mm/Hg at 25 °C) and with a low enthalpy of vaporisation (> 20 KJoule/mol): their concentration in the alveolar air should be so low that we cannot detect them with our equipment.

Next we illustrate the compounds with the mentioned MW and which corresponded to the criteria above reported: only ethylphosphane (CAS 593-68-0), had the right inclusion criteria for compounds with MW 62 Da.

Among the products with MW of 71 Da, the possible presence in the alveolar air concerned the Formyl cyanate (CAS 471-47-0) and the 1,2,3,4-Oxatriazole. (ChemSpider ID: 10609508).

The majority of the compounds with MW of 74 Da had a boiling point above 150 °C, and just four satisfied the other criteria reported: methallylamine [EINECS 220-724-2], N-ethylethanimine (CAS 1190-79-0), N-ethylethenamine (ChemSpider ID: 9415459), 2,3-Dimethylaziridine (ChemSpider ID: 10295849). Three compounds with MW of 89 Da showed the required parameters: 1-nitropropane (CAS 108-03-2), 2-nitropropane (CAS 79-46-9) and 2-(Dimethyl amino)ethanol (CAS 108-01-0). Many molecules with MW of 112 Da are of artificial origin; among those which satisfy our inclusion criteria we find 2,3-Diamino-1,3-butadiene-1,4-dione (ChemSpider ID: 17269477) and 1,1-diisocyanatoethane (ChemSpider ID: 10122761).

Further studies will be necessary to find out which molecules, among those we have pointed out (even if not-named), could be considered the most specific and useful markers in the detection of the pancreatic adenocarcinoma.

With the involvement of new cases of pancreatic neoplasm we ran the model for the validation test that didn’t confirm the same sensitivity, nevertheless the results were good.

The predictive model reaches 100% sensitivity and a good specificity which can be recalibrated, for clinical or epidemiological studies in order to have higher specificity values with an acceptable sensitivity. Other requirements for a good analysis, met by this method, are: speed, security, minimal invasiveness and low costs. Indeed, it is a very quick test. After a short explanation about the importance of a deep inhalation, the sample can be obtained in the time of an exhalation.

Certainly, in “preliminary performance studies” such as this one, each attempt to identify illness indicators can prove vain. Since it could point out some altered elements in a productive phase, it cannot be used in an early phase [41]**.** The appearance of clinical manifestations in this kind of tumour can be both early and late, depending on the region of the pancreas concerned. Therefore, it is not possible at the first hospitalization to speak of neoplasm as being at the early stages or as being very advanced. The evolution of the pancreatic cancer and the inadequate currently available instruments for early diagnosis in this field persuaded us to study it through the analysis of the alveolar air. Oxidative stress is a possible causal factor for pancreatic cancer [42, 43] and is hypothetically able to alter the concentration of volatile compound exhaled. At present, our intuition is that in some way it is not the substances taken one by one that are so important for the detection of the pancreatic cancer, but the “movement” of a group of different molecules that determines the prediction of pathology. To the best of our knowledge, this is the first study which addresses the possibility of identifying patients suffering from PDA by the VOC profile of alveolar air.

## Conclusions

Predictive models, based on VOCs profiles in alveolar air, obtained from the use of high precision instruments and advanced statistical methods, could contribute to the diagnosis of such a cryptic kind of neoplasm with high sensitivity and specificity. We reach 100% sensitivity with a specificity of 84%. The analysis of alveolar air, in view of the first results achieved, is undoubtedly a very promising test for detection of pancreatic ductal adenocarcinoma. The integration of VOCs profiles and blood specific markers could further improve the detection of different kind of neoplasms and other pathologies.

## Additional file

Additional file 1: Environmental and alveolar concentrations of measured volatile organic compounds. The file contains all raw data about environmental (sheet 1) and alveolar (sheet 2) concentrations of measured products with the sample codes and the main parameters (sex, age and case-control classification) of enrolled subjects. Red printed VOCs were directly calibrated for the mass spectrometer analysis. (XLSX 417 kb)
